# Mortality and Severity in COVID-19 Patients on ACEIs and ARBs—A Systematic Review, Meta-Analysis, and Meta-Regression Analysis

**DOI:** 10.3389/fmed.2021.703661

**Published:** 2022-01-10

**Authors:** Romil Singh, Sawai Singh Rathore, Hira Khan, Abhishek Bhurwal, Mack Sheraton, Prithwish Ghosh, Sohini Anand, Janaki Makadia, Fnu Ayesha, Kiran S. Mahapure, Ishita Mehra, Aysun Tekin, Rahul Kashyap, Vikas Bansal

**Affiliations:** ^1^Department of Anesthesiology and Critical Care Medicine, Mayo Clinic, Rochester, MN, United States; ^2^Dr. Sampurnanand Medical College and Hospital, Jodhpur, India; ^3^Department of Internal Medicine, Islamic International Medical College, Rawalpindi, Pakistan; ^4^Department of Gastroenterology and Hepatology, Rutgers Robert Wood Johnson School of Medicine, New Brunswick, NJ, United States; ^5^Department of Emergency Medicine, Trinity West Medical Center, Steubenville, OH, United States; ^6^Department of Gastroenterology and Hepatology, Mayo Clinic, Rochester, MN, United States; ^7^Patliputra Medical College and Hospital, Dhanbad, India; ^8^GMERS Medical College and Hospital, Vadodara, India; ^9^Department of Internal Medicine, Services Institute of Medical Sciences, Lahore, Pakistan; ^10^Department of Plastic Surgery, KAHER J. N. Medical College, Belgaum, India; ^11^Department of Internal Medicine, North Alabama Medical Center, Florence, AL, United States; ^12^Department of Pulmonary and Critical Care Medicine, Mayo Clinic, Rochester, MN, United States

**Keywords:** COVID-19, Angiotensin inhibitors, ACEI, ARB, mortality, severity, meta-analysis, meta-regression

## Abstract

**Purpose:** The primary objective of this systematic review is to assess association of mortality in COVID-19 patients on Angiotensin-converting-enzyme inhibitors (ACEIs) and Angiotensin-II receptor blockers (ARBs). A secondary objective is to assess associations with higher severity of the disease in COVID-19 patients.

**Materials and Methods:** We searched multiple COVID-19 databases (WHO, CDC, LIT-COVID) for longitudinal studies globally reporting mortality and severity published before January 18th, 2021. Meta-analyses were performed using 53 studies for mortality outcome and 43 for the severity outcome. Mantel-Haenszel odds ratios were generated to describe overall effect size using random effect models. To account for between study results variations, multivariate meta-regression was performed with preselected covariates using maximum likelihood method for both the mortality and severity models.

**Result:** Our findings showed that the use of ACEIs/ARBs did not significantly influence either mortality (OR = 1.16 95% CI 0.94–1.44, *p* = 0.15, *I*^2^ = 93.2%) or severity (OR = 1.18, 95% CI 0.94–1.48, *p* = 0.15, *I*^2^ = 91.1%) in comparison to not being on ACEIs/ARBs in COVID-19 positive patients. Multivariate meta-regression for the mortality model demonstrated that 36% of between study variations could be explained by differences in age, gender, and proportion of heart diseases in the study samples. Multivariate meta-regression for the severity model demonstrated that 8% of between study variations could be explained by differences in age, proportion of diabetes, heart disease and study country in the study samples.

**Conclusion:** We found no association of mortality or severity in COVID-19 patients taking ACEIs/ARBs.

## Introduction

SARS-CoV-2 originated in Wuhan, China, in December 2019 and has spread to every major country in the world and was subsequently declared a pandemic on March 11, 2020 (1). As of April 29th, 2021, there were 150,088,112 positive patients worldwide; and 3,161,337 of these patients were reported to be deceased because of SARS-CoV-2 (2). The case fatality rate of SARS-CoV-2 in the U.S. is 1.8% as per COVID-19 Dashboard by the Center for Systems Science and Engineering (CSSE) at Johns Hopkins University (2). However, the role of different medications and comorbidities has been elicited in recent articles.

The SARS-CoV-2 disease varies from mild to fulminant in reference to several risk variables contributing to a poor prognosis (3–5). While the virus significantly impacts the respiratory tract, other metabolic systems have been involved in numerous case studies and systematic reviews (6–14). Thorough awareness of the risks, pathogenesis, and predisposing factors together with the important aspects in the diagnosis is of paramount importance in order to direct decision-making for acute care and mitigate mortality of COVID-19 (15–18).

Severe acute respiratory syndrome coronavirus 2 SARS-CoV-2 uses the receptor angiotensin-converting enzyme (ACE) 2 for entry into target cells, and it was reported that both Angiotensin-converting enzyme inhibitors (ACEIs) and angiotensin receptor blockers (ARBs) could increase the mRNA expression of cardiac ACE2 receptors (19, 20). However, controversy about the novel use of Renin-angiotensin system (RAS) blockers has been raised amid this SARS-CoV-2 pandemic. The explanation behind this controversy arises from the very fact that it shares the target receptor site with ACEIs and ARBs, which can cause the upregulation of ACE2 receptors (21). ACE2 is additionally the notable cellular surface receptor and a necessary entry point for SARS-CoV-2 into the target cell (22, 23). As cardiovascular diseases and their therapy affects ACE2 levels, it plays an integral part in consideration of infectivity and outcomes of SARS-CoV-2 (20). It needs to be imperatively determined whether treatment or disease-induced up-regulation of ACE2 impacts the trajectory of SARS-CoV-2 (19). ACEIs/ARBs are often used to treat hypertension, which is the most common comorbidity associated with SARS-CoV-2 (20, 24). As there is no clinical evidence, major international cardiology societies recommend continuing the use of ACEIs and ARBs in SARS-CoV-2 patients (25).

Due to limited literature on the influence of ACE inhibitors and ARBs in COVID-19 patients, we systematically reviewed the relevant medical literature. We performed a meta-analysis and meta-regression to investigate the association of ACEIs and ARBs used in COVID-19 and its effect on the mortality rate and severity of COVID-19.

## Materials and Methods

We have presented this review according to the Preferred Systematic Reviews and Meta-Analysis Reporting Items guidelines for documenting analysis (26). We attempted to register this systematic review but opted against it because it was taking an extended amount of time due to the large number of COVID-19-related literature being submitted.

### Search Strategy

**Figure 1:**
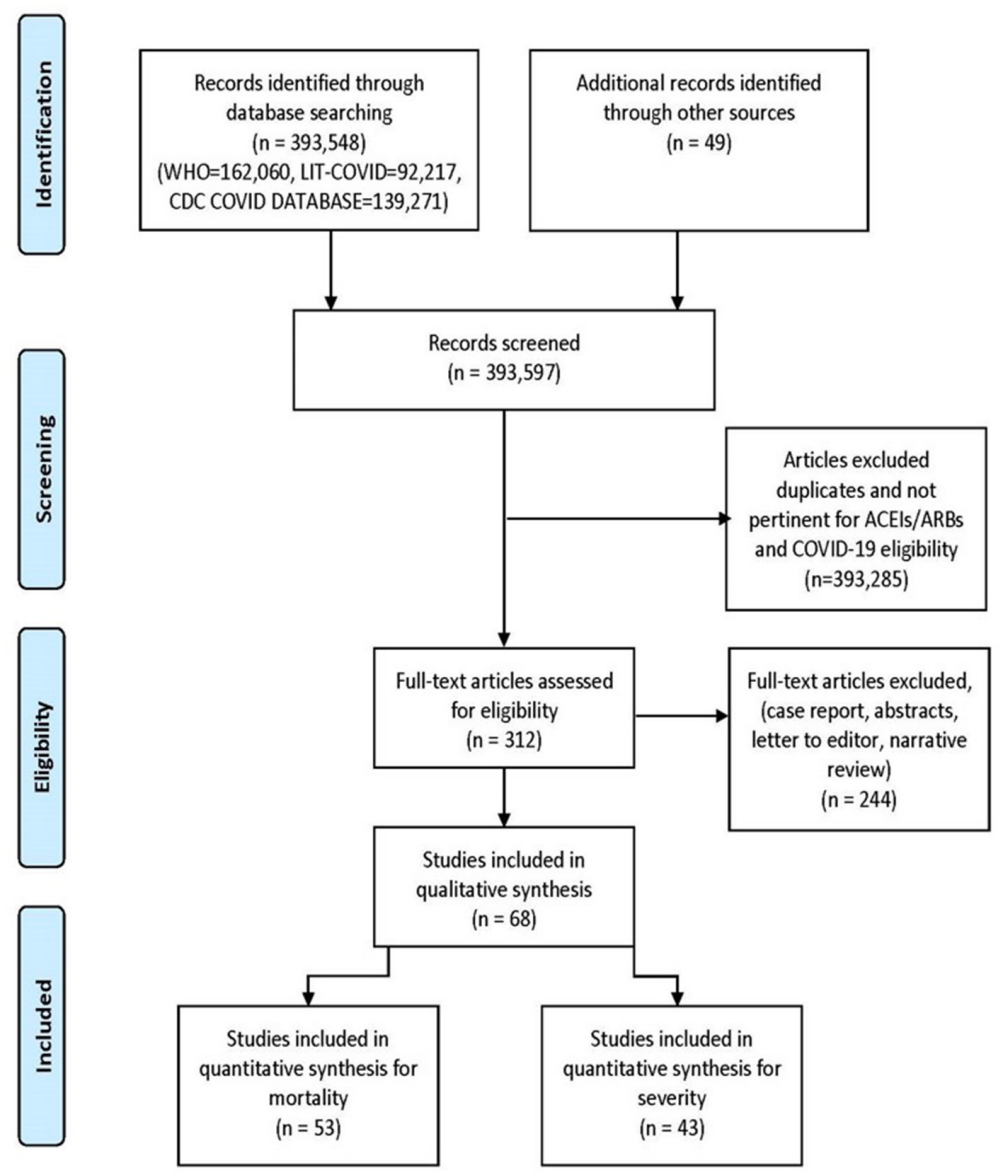
PRISMA flow diagram.

We searched WHO COVID-19 Global research database, Lit-COVID (27), CDC Database of COVID-19 Research including PubMed, Embase, Scopus, Science Web, and Cochrane Central Controlled Trials Registry. The MedRxiv and SSNR preprint servers were also scanned. The searches were performed from December 2019 and revised till January 18th, 2021. The search approach and design can be found in Figure 1. Studies from all around the world were included, there were no language barriers. In an attempt to discover further eligible studies, we manually searched the reference lists of the included studies and the relevant literature. We also scanned the ClinicalTrials.gov registry for completed, as well as in-progress randomized controlled trials (RCTs).

### Eligibility Criteria

Observational studies that met all the following criteria were included: (1) study design: case-control, case-crossover, self-controlled case series (SCCS) or cohort study; (2) reported antihypertensive treatment: ACEI/ARB use vs. non-ACEI/ARB use; (3) outcomes: the incidence of COVID-19 mortality or severity; (4) adequate data were used to extract the risk estimates if the adjusted data were not provided in the publication. Studies focusing on patients <18 years of age, focusing on pregnant females, and limited to particular comorbidities and organ dysfunctions were excluded to avoid selection bias. We also excluded case reports, Editorials, correspondences, conference abstracts and commentary articles were excluded in our study. When information was incomplete in the publication, attempts were made to contact the study investigators to obtain missing information.

### Study Selection

Three authors (RS, SR, and HK) downloaded all articles from electronic search to EndNote X9 (28), as well as duplicates were eliminated. Titles and abstracts were autonomously evaluated by authors (AT, FA, HK, JM, KM, PG, RS, SA, and SR) to identify and assess key articles. Further, authors (FA, HK, JM, KM, PG, RS, SA, and SR) independently reviewed the entire manuscript and registered justification for the exclusion. Any discrepancies were addressed by arbitration.

### Outcome

All-cause mortality in the COVID-19 affected patient was the chief outcome, while severity of disease was the secondary outcome. We defined severity as the need for ICU admission or the need for Mechanical Ventilation. If both severities were given in the article, then we collect the highest amount of data for respective events as the severity for COVID-19.

### Data Extraction

We included all observational studies that satisfied our inclusion criteria. Using a standardized data extraction method, the authors (FA, HK, JM, KM, PG, RS, SA, and SR) extracted information from each survey independently; any conflict was resolved by consensus. The following dataset points were extracted: First author name, cases on ACEI-ARB, total COVID positive patients, country of study, study design, hypertension proportion, diabetes proportion, heart disease proportion, eligibility criteria, Median age, gender (female sex proportion), comorbidities, use of ACEIs or ARBs and primary and secondary outcomes (mortality and severity). Unadjusted and adjusted impact measurements were also extracted where appropriate. The majority of papers differentiated between cases (use of ACEIs and ARBS) and controls (ACEIs/ARBS not used). However, we manually obtained the division in a few publications.

### Statistical Analysis

The meta-analysis specifically included longitudinal and cross-sectional studies comparing the effects of COVID-19 in subjects who were on ACEIs/ARBs at the time of infection with those who were not. Meta-analysis was performed first for studies reporting mortality of patients in both groups, followed by that for studies reporting severity of disease assuming independence of results for studies that reported both. Due to anticipated heterogeneity, summary statistics were calculated using a random-effects model. This model accounts for variability between studies as well as within studies. In all cases, meta-analyses were performed using the Mantel-Haenszel method for dichotomous data to estimate pooled odds ratios (OR) and statistical heterogeneity was assessed using *Q*-value and *I*^2^ statistics. The meta-analysis and meta-regression was done with the Comprehensive Meta-Analysis software package (Biostat, Englewood, NJ, USA) (29). We included the region of study in meta-regression model to find out whether Asian studies, which were dated earlier than studies from the rest of the world, contributed disproportionately to the significance of results. This helped rule out location and pipeline biases.

To explore differences between studies that might be expected to influence the effect size, we performed random effects (maximum likelihood method) univariate and multivariate meta-regression analyses. The potential sources of variability defined were median age of study sample, proportion of subjects of female sex, proportion of diabetics and proportion with heart diseases. Covariates were selected for further modeling if they significantly (*P* < 0.05) modified the association between mortality or severity in the COVID19 infected and treatment with ACEIs/ARBs. Two models were created, one for mortality and the other for severity of disease as outcomes. Subsequently, preselected covariates were included in a manual backward and stepwise multiple meta-regression analysis with *P* = 0.05 as a cutoff point for removal. *P* < 0.05 (*P* < 0.10 for heterogeneity) was considered statistically significant. All meta-analysis and meta-regression tests were 2-tailed.

### Risk of Bias Assessment

The Newcastle-Ottawa (NOS) scale (30) was used for measuring the risk of bias in cohort and case-control studies (Tables 2A,B). The following classes were rated per study: low bias risk (9 points), moderate bias risk (5–7 points), and high bias risk (0-4 items). For a cross-sectional study, we used the modified version of NOS, assigning the study in the following groups: Low risk of bias (8–10), moderate risk (5–7), high risk of bias (0–4). Three reviewers (RS, SR, and PG) evaluated the likelihood of bias independently, and any conflict was resolved by consensus.

## Results

The initial library search identified potentially relevant citations from WHO Global Research Database, CDC COVID-19 Research Articles Downloadable Database, and LitCovid PubMed database comprised of 393,597 articles. Subsequently, 393,285 articles were removed because of unclear evidence and non-relevance to the objective of the manuscript. Out of the remaining 312 articles, a total of 244 articles consisting of case reports, abstracts, letter to editor, and narrative reviews were excluded. Thus, 68 studies (31–98) were included in their entirety as shown in Table 1. The PRISMA flow chart is shown in Figure 1.

**Table 1:** Study characteristics.

**First author**	**Publication status**	**Type of study**	**Outcome data**	**Country of study**	**Median age**	**Female sex Proportion**	**Diabetes proportion**	**Heart disease proportion**	**Hypertension proportion (in percentage)**	**High severity (case)**	**High severity sample size (case)**	**High Severity (control)**	**High severity Sample size (control)**	**Mortality (case)**	**Sample size (case)**	**Mortality (control)**	**Sample size (control)**
Alexandre et al.	Peer-reviewed	Retrospective Cohort study	All-cause mortality and severity	UK	61	39.76	28.24	17.57	48.7	17	117	12	230	17	117	12	230
Anzola et al.	Peer-reviewed	Prospective study	Severity	Italy	65	39	14	21	51	35	140	16	291	N/A	N/A	N/A	N/A
Ashraf et al.	Pre-print	Cross sectional	Severity	Iran	58	35.4	26	19	26	4	19	11	81	N/A	N/A	N/A	N/A
Bae et al.	Peer-reviewed	Cohort	All-cause mortality and severity	USA	52	51.2	26.2	8.9	25.4	20	78	54	512	1	78	5	512
Baker et al.	Pre-print	Cohort	All-cause mortality	UK	75	46	26.6	20.6	42.1	N/A	N/A	N/A	N/A	17	78	63	233
Banerjee et al.	Peer-reviewed	Cohort	All-cause mortality and severity	UK	61.5	42.8	43.8	N/A	100	1	1	1	6	1	1	0	6
Bauer et al.	Peer-reviewed	Case–control study	All-cause mortality	USA	54.7	63	17	7	36	N/A	N/A	N/A	N/A	77	230	198	1,219
Bean et al.	Peer-reviewed	Case-Control	All-cause mortality and severity	UK	69.235	42.8	34.8	13.3	100	127	399	288	801	106	399	182	801
Benelli et al.	Pre-print	Cohort	All-cause mortality	Italy	66.8	33.4	16.3	22.6	46.95	N/A	N/A	N/A	N/A	25	110	47	301
Braude et al.	Peer-reviewed	Multicenter observational study	All-cause mortality	UK+ Italy	74	40.9	27.1	21.8	51.5	N/A	N/A	N/A	N/A	106	392	257	979
Bravi et al.	Peer-reviewed	Case-Control	Severity	Italy	58	52.3	12.1	16.1	33.9	267	450	69	93	N/A	N/A	N/A	N/A
Cariou et al.	Peer-reviewed	Cohort	All-cause mortality and severity	France	69.8	35.1	88.5	11.6	77.2	232	737	150	580	92	737	48	580
Cetinkal et al.	Peer-reviewed	Retrospective single center study	All-cause mortality and severity	Turkey	68.7	49.57	40.4	51	100	45	201	27	148	29	201	20	148
Chaudri et al.	Peer-reviewed	Single center cohort study	All-cause mortality and severity	USA	62	34	24.6	28.6	44.3	59	80	22	220	5	80	25	220
Chen Ming et al.	Pre-print	Case control	All-cause mortality	China	28.85	50.4	11.3	16.2	33.33	N/A	N/A	N/A	N/A	3	11	28	112
Chen Yuchen et al.	Peer-reviewed	Cohort	All-cause mortality	China	67.25	33	100	N/A	100	N/A	N/A	N/A	N/A	4	32	10	39
Choi et al.	Pre-print	Case control	All-cause mortality and severity	South Korea	66.5	57.2	44.9	N/A	100	34	892	55	625	42	892	69	625
Conversano et al.	Peer-reviewed	Cohort	All-cause mortality	Italy	65	31.4	14.6	4.7	50.26	N/A	N/A	N/A	N/A	48	69	101	122
De Spiegele er et al.	Peer-reviewed	Cohort	Severity	Europe	85.9	67	18.1	N/A	25.3	6	30	31	124	N/A	N/A	N/A	N/A
Covino et al.	Peer-reviewed	Retrospective study	All-cause mortality and severity	Australia	74	34.3	13.2	42.1	100	38	51	82	115	58	111	22	55
Desai et al.	Peer-reviewed	Retrospective study	All-cause mortality	Italy	64.8	33.9	20	27.1	43.1	N/A	N/A	N/A	N/A	49	154	72	421
Felice et al.	Peer-reviewed	Cohort	All-cause mortality and severity	Italy	72	35.4	25.5	18	100	21	82	25	51	15	82	18	51
Zhou feng et al.	Peer-reviewed	Cohort	All-cause mortality	China	66	49.9	N/A	N/A	100	N/A	N/A	N/A	N/A	70	906	272	1812
Fosbol et al.	Peer-reviewed	Cohort	All-cause mortality and severity	Denmark	62	52	9	8.5	18.8	203	895	373	3585	181	895	297	3585
Golpe et al.	Peer-reviewed	Cohort	Severity	Spain	70.4	54.1	9.8	N/A	29.1	48	69	73	88	N/A	N/A	N/A	N/A
Guner et al.	Peer-reviewed	Cohort	Severity	Turkey	50.6	40.5	13.5	23.6	23.4	21	65	29	167	N/A	N/A	N/A	N/A
Genet et al.	Peer-reviewed	Retrospective observational study	All-cause mortality and severity	USA	86.3	36.19	10.45	12.6	33.5	14	63	52	138	14	63	52	138
Guo et al.	Peer-reviewed	Cross sectional	All-cause mortality	China	58	51.3	15	15.5	32.62	N/A	N/A	N/A	N/A	7	19	36	168
Hakeam et al.	Peer-reviewed	Multicenter prospective cohort	All-cause mortality and severity	Saudi Arabia	60.8	40.5	63.3	32.2	93.7	69	245	33	93	15	69	7	33
Huh et al.	Peer-reviewed	Retrospective cohort study	Severity	Korea	47.1	59.54	16.06	7.83	21.47	248	877	630	6,464	N/A	N/A	N/A	N/A
Huang et al.	Peer-reviewed	Cohort	All-cause mortality	China	60.185	55	8	2	100	N/A	N/A	N/A	N/A	0	20	3	30
Kim Ju Hwan et al.	Peer-reviewed	Retrospective cohort study	All-cause mortality and severity	South Korea	62.8	49.79	62.69	53.89	22.6	23	628	28	608	23	628	28	608
Ip et al.	Pre-print	Cross sectional	All-cause mortality	USA	N/A	N/A	N/A	N/A	52.5	N/A	N/A	N/A	N/A	137	460	262	669
Jurado et al.	Pre-print	Cross sectional	Severity	Spain	63.2	40.6	23.8	N/A	52.4	56	92	135	198	N/A	N/A	N/A	N/A
Jung et al.	Peer-reviewed	Cohort	All-cause mortality	South Korea	44.6	56	17	5	22.34	N/A	N/A	N/A	N/A	33	377	51	1577
Kim Lindsay et al.	Peer-reviewed	Cohort	All-cause mortality	USA	62	46.8	32.9	34.6	57.32	N/A	N/A	N/A	N/A	105	573	530	3214
Lafaurie et al.	Peer-reviewed	Cohort study	All-cause mortality and severity	UK	74	77.56	12.92	12.9	38.78	36	73	14	36	9	73	6	36
Lee et al.	Pre-print	Cohort	All-cause mortality	South Korea	44.36	77.56	17	6.7	19	N/A	N/A	N/A	N/A	50	977	62	7,289
Li et al.	Peer-reviewed	Cross sectional	All-cause mortality and severity	China	66	47.8	35	35.07	30.73	57	115	116	247	21	115	56	247
Liu et al.	Pre-print	Cross sectional	Severity	China	65.2	44.9	N/A	N/A	15.26	7	22	31	56	N/A	N/A	N/A	N/A
Mancia et al.	Peer-reviewed	Cross sectional	Severity	Italy	68	36.7	N/A	30.1	58	364	2,896	253	3,376	N/A	N/A	N/A	N/A
Lim et al.	Peer-reviewed	Retrospective cohort study	All-cause mortality and severity	South Korea	67	46.15	25.38	10	40	14	30	22	100	14	30	22	100
Mehta et al.	Peer-reviewed	Cohort	All-cause mortality	USA	58.5	49.9	41.1	19.9	11.6	N/A	N/A	N/A	N/A	8	211	34	1,494
Meng et al.	Peer-reviewed	Cross sectional	All-cause mortality and severity	China	64.5	42.8	30.95	N/A	10	4	17	12	25	0	17	1	25
Lopez-Otero et al.	Peer-reviewed	Cohort	All-cause mortality	Spain	64.05	56	13	21.2	100	N/A	N/A	N/A	N/A	11	211	27	755
Oussalah et al.	Peer-reviewed	Retrospective longitudinal cohort study	All-cause mortality and severity	France	65	39	29	29	50	20	44	34	105	10	43	9	104
Peng et al.	Peer-reviewed	Cross sectional	All-cause mortality and severity	China	31	52.68	26	66.3	82.14	3	22	13	90	4	22	13	90
Rezel-Potts et al.	Peer-reviewed	Case control study with additional cohort analysis	All-cause mortality	UK	62	60	13	14	26.8	N/A	N/A	N/A	N/A	254	2712	667	14154
Reynolds et al.	Peer-reviewed	Cross sectional	Severity	USA	32	58.5	39.7	16.1	34.59	587	2,401	608	2,393	N/A	N/A	N/A	N/A
Rhee et al.	Pre-print	Cohort	Severity	South Korea	62.375	46.5	N/A	18.5	68.02	13	327	21	505	N/A	N/A	N/A	N/A
Rentsch et al.	Pre-print	Cohort	All-cause mortality	USA	66.1	4.6	44.4	27.9	71.96	N/A	N/A	N/A	N/A	11	255	6	324
Richardson et al.	Peer-reviewed	Cross sectional	All-cause mortality and severity	USA	63	39.7	59.8	33.8	53.08	87	413	141	953	130	413	254	953
Rodilla et al.	Peer-reviewed	Cross-sectional, observational, retrospective multicenter study,	All-cause mortality	Spain	67.5	42.6	19.1	4	50.9	N/A	N/A	N/A	N/A	1,180	4,238	1,452	7,988
Rosenthal et al.	Peer-reviewed	Retrospective cohort study	All-cause mortality	USA	57	50.1	27.9	22.09	46.7	N/A	N/A	N/A	N/A	345	3,162	7,010	34,545
Rossi et al.	Pre-print	Cohort	All-cause mortality	Italy	31.6	49.9	12	5.8	16.2	N/A	N/A	N/A	N/A	108	818	109	1835
Soleimani et al.	Peer-reviewed	Retrospective observational study	All-cause mortality and severity	USA	66.4	16.5	0.18	13.99	39.93	91	122	91	132	33	122	35	132
Tan et al.	Peer-reviewed	Case control	All-cause mortality and severity	China	67.25	49	28	18	100	27	31	60	69	0	31	11	69
Lam et al.	Peer-reviewed	Retrospective single center study	All-cause mortality and severity	USA	70.5	9.89	8.96	8.24	31.37	65	335	55	279	58	335	62	279
Wang et al.	Peer-reviewed	Cross sectional	All-cause mortality	China	64	48	18.6	11.6	41.86	N/A	N/A	N/A	N/A	30	62	103	282
Xian et al.	Peer-reviewed	Case-control	All-cause mortality	China	57.7	45.5	10	9.1	32.7	N/A	N/A	N/A	N/A	2	15	5	21
Xiulan et al.	Peer-reviewed	Retrospective single-center case series	All-cause mortality and severity	China	65.39	53.5	22	16	100	31	74	38	83	5	74	1	83
Yan et al.	Pre-print	Cohort	Severity	China	48.75	48.9	10	2.62	22.46	52	256	76	354	N/A	N/A	N/A	N/A
Yun Feng et al.	Peer-reviewed	Case-control	Severity	China	53	43.1	10.3	8	25.8	4	33	36	80	N/A	N/A	N/A	N/A
Zeng et al.	Pre-print	Cross sectional	All-cause mortality and severity	China	66.5	50.6	56	41.3	27.37	15	28	15	47	2	28	5	47
Zhang et al.	Peer-reviewed	Cohort	All-cause mortality	China	64	46	21.3	29.09	100	N/A	N/A	N/A	N/A	7	188	92	940
Zhichao Feng et al.	Pre-print	Cohort	Severity	China	47	49.6	8	4	14.5	1	16	16	49	N/A	N/A	N/A	N/A
Zhong et al.	Peer-reviewed	Retrospective observational study	All-cause mortality and severity	UK	66.3	55.5	32.5	16.6	100	6	37	15	89	6	37	15	89

**Table 2:** New Castle Ottawa scale.

**(A) COHORT STUDIES**									
**Study**	**Selection**				**Comparability**	**Outcome**			**Total (maximum = 9)**
	**Representa -tiveness of the exposed cohort**	**Selection of the non-exposed cohort**	**Ascertainment of the exposure**	**Outcome status at start of study**		**Assessment of the outcome**	**Length of follow-up**	**Adequacy of follow-up**	
Alexandre et al.	*	*	*	*	*	*	*	*	8
Anzola et al.	*	*	*	*	**	*	*	*	9
Bae et al.	*	*	*	*	**	*	*	*	9
Baker et al.	*	*	*	*	-	*	*	*	7
Baneerje et al.	*	*	*	*	*	*	*	*	8
Beanet al.	*	*	*	*	-	*	*	*	7
Benelli et al.	*	*	*	*	-	*	*	*	7
Braude et al.	*	*	*	*	*	*	*	*	8
Bravi et al.	*	*	*	*	*	*	*	*	8
Cariou et al.	*	*	*	*	*	*	*	*	8
Cetinkal et al.	*	*	*	*	**	*	*	*	9
Chaudri et al.	*	*	*	*	*	*	*	*	8
Conversano et al.	*	*	*	*	**	*	*	*	9
Covino et al.	*	*	*	*	*	*	*	*	8
De Spiegeleer et al.	*	*	*	*	**	*	*	*	9
Desai et al.	*	*	*	*	*	*	*	*	8
Feng zhou et al.	*	*	*	*	-	*	-	*	6
Fosbol et al.	*	*	*	*	**	*	-	-	7
Genet et al.	*	*	*	*	-	*	*	*	7
Golpe et al.	*	*	*	*	*	*	*	*	8
Guner et al.	*	*	*	*	*	*	*	*	8
Hakeam et al.	*	*	*	*	**	*	*	*	9
Huang et al.	*	*	*	*	-	*	*	*	7
Huh et al.	*	*	*	*	**	*	*	*	9
Hwan Kim et al.	*	*	*	*	**	*	*	*	9
Jung et al.	*	*	*	*	**	*	*	*	9
Lafaurie et al.	*	*	*	*	**	*	*	*	9
Lee et al.	*	*	*	*	**	*	*	*	9
Lee et al.	*	*	*	*	**	*	*	*	9
Lim et al.	*	*	*	*	*	*	*	*	8
Mehta et al.	*	*	*	*	-	*	*	*	7
Otero et al.	*	*	*	*	*	*	*	*	8
Oussalah et al.	*	*	*	*	**	*	*	*	9
Rentsch et al.	*	*	*	*	**	*	*	*	9
Rhee et al.	*	*	*	*	**	*	*	*	9
Rodilla et al.	*	*	*	*	-	*	-	*	6
Rosenthal et al.	*	*	*	*	*	*	*	*	8
Rossi et al.	*	*	*	*	*	*	*	*	8
Soleimani et al.	*	*	*	*	-	*	*	*	7
Lam et al.	*	*	*	*	*	*	*	*	8
Xiulan et al.	*	*	*	*	-	*	*	*	7
Yan et al.	*	*	*	*	*	*	*	*	8
Yang et al.	*	*	*	*	-	*	*	*	7
Yuchen et al.	*	*	*	*	-	*	*	*	7
Zhang et al.	*	*	*	*	**	*	*	*	9
Zhichao Feng et al.	*	*	*	*	*	*	*	*	8
Zhong et al.	*	*	*	*	*	*	*	*	8
Zhou et al.	*	*	*	*	*	*	*	*	8
**(B) CASE CONTROL STUDIES**									
**Study**	**Selection**				**Comparability**			**Total (maximum** **=** **10)**
	**Representativeness**	**Sample size**	**Non-respondents**	**Ascertainment of the exposure**		**Assessment of the outcome**	**Statistical test**	
Ashraf, 2020	*	-	*	*	-	**	-	5
Bauer et al.	*	*	*	*	**	**	*	9
Chen, 2020	*	-	*	*	-	**	-	5
Choi, 2020	*	*	*	*	*	**	*	8
Felice, 2020	*	-	*	*	-	**	-	5
Guo, 2020	*	-	*	*	-	**	-	5
Ip, 2020	*	*	*	*	-	**	-	6
Jurado, 2020	*	*	*	*	-	**	-	6
Li, 2020	*	*	*	*	-	**	-	6
Liu, 2020	*	-	*	*	*	**	*	7
Mancia, 2020	*	*	*	*	-	**	-	6
Meng, 2020	*	-	*	*	-	**	-	5
Peng, 2020	*	-	*	*	-	**	-	5
Potts et al.	*	-	*	*	*	**	*	7
Reynolds, 2020	*	*	*	*	**	**	*	9
Richardson, 2020	*	*	*	*	-	**	-	6
Tan, 2020	*	*	*	*	-	**	-	6
Wang, 2020	*	-	*	*	-	**	-	5
Yun Feng, 2020	*	-	*	*	-	**	-	5
Zeng, 2020	*	-	*	*	-	**	-	5

* One point is designated to that particular entity; **Two point designated; −, zero point designated.

### Study Characteristics of Included Studies

A total of 53 studies (31–83) were included for meta-analysis for the primary outcome i.e., mortality. In total, these consisted of 112,468 subjects with 16,363 mortality events. Median age for all studies was 64.9 (60.9–67.2) with average 47.3% females (Table 1). Of the comorbidities considered, 25.4% were diabetics, 16.6% had heart diseases overall. Similarly, a total of forty three studies (31, 33, 35, 38–40, 43, 45, 47–49, 51, 55, 57–59, 61–63, 66–68, 71, 75, 76, 78, 79, 81, 84–98) were included for meta-analysis for the secondary outcome i.e., severity of disease. These had a combined sample size of 37,914 with 6,985 patients reaching the endpoint of high disease severity. The median age was 65.2 (60.8–68.7) and 46.2% were females, 25.6% were diabetics, 17.1% had heart diseases overall in this cohort (Table 1).

### Meta-Analysis for Mortality Outcome

**Figure 2:**
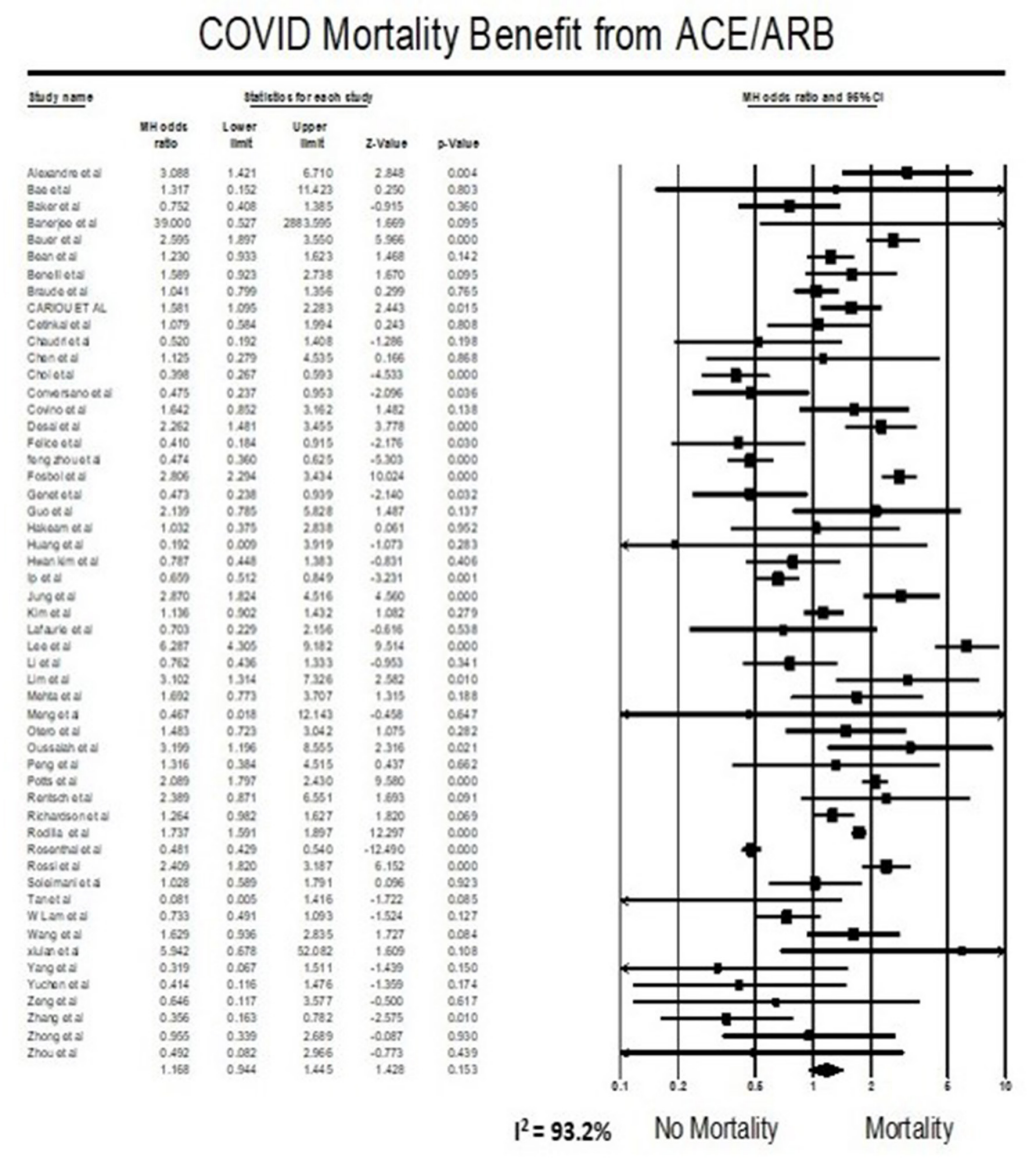
Forest plot for association of ACE/ARBs on mortality in COVID19 patients.

Meta-analysis findings showed that being on ACEIs/ARBs did not have an association with mortality from COVID 19 infections compared to not being on ACEIs/ARBs (OR = 1.17, 95% CI 0.94–1.45, *p* = 0.15). Heterogeneity was very high with *I*^2^ = 93.2% (Figure 2).

### Meta-Analysis for Severity Outcome

**Figure 3:**
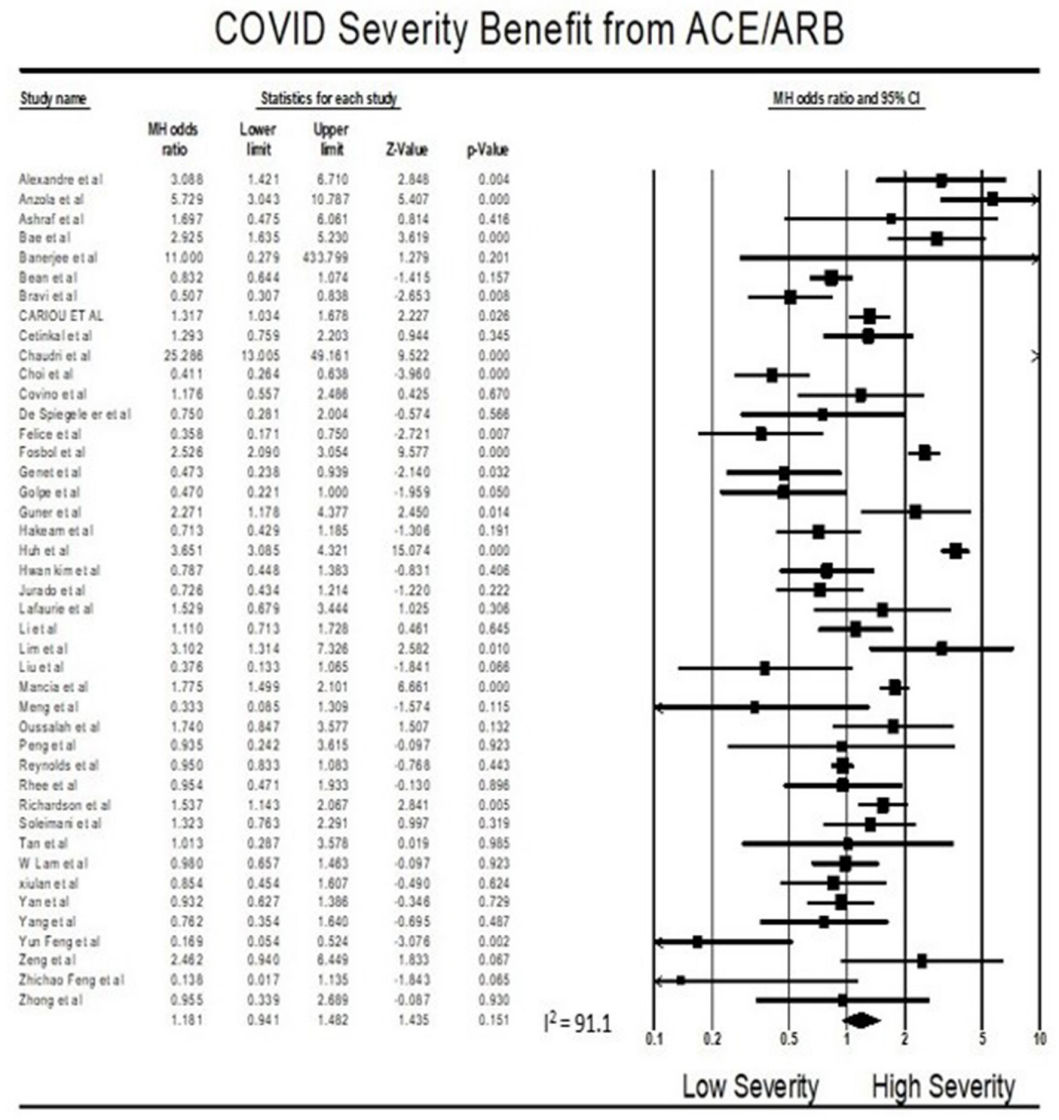
Forest plot for association of ACE/ARBs on severity in COVID19 patients.

Findings from the meta-analysis showed that being on ACEI/ARBs did not have an association with severity from COVID 19 infections compared to not being on ACEIs/ARBs (OR = 1.18, 95% CI 0.94–1.48, *p* = 0.15). Heterogeneity was very high with *I*^2^ = 91.1% (Figure 3).

### Multivariate Meta-Regression Model for Mortality Outcome

**Figure 4:**
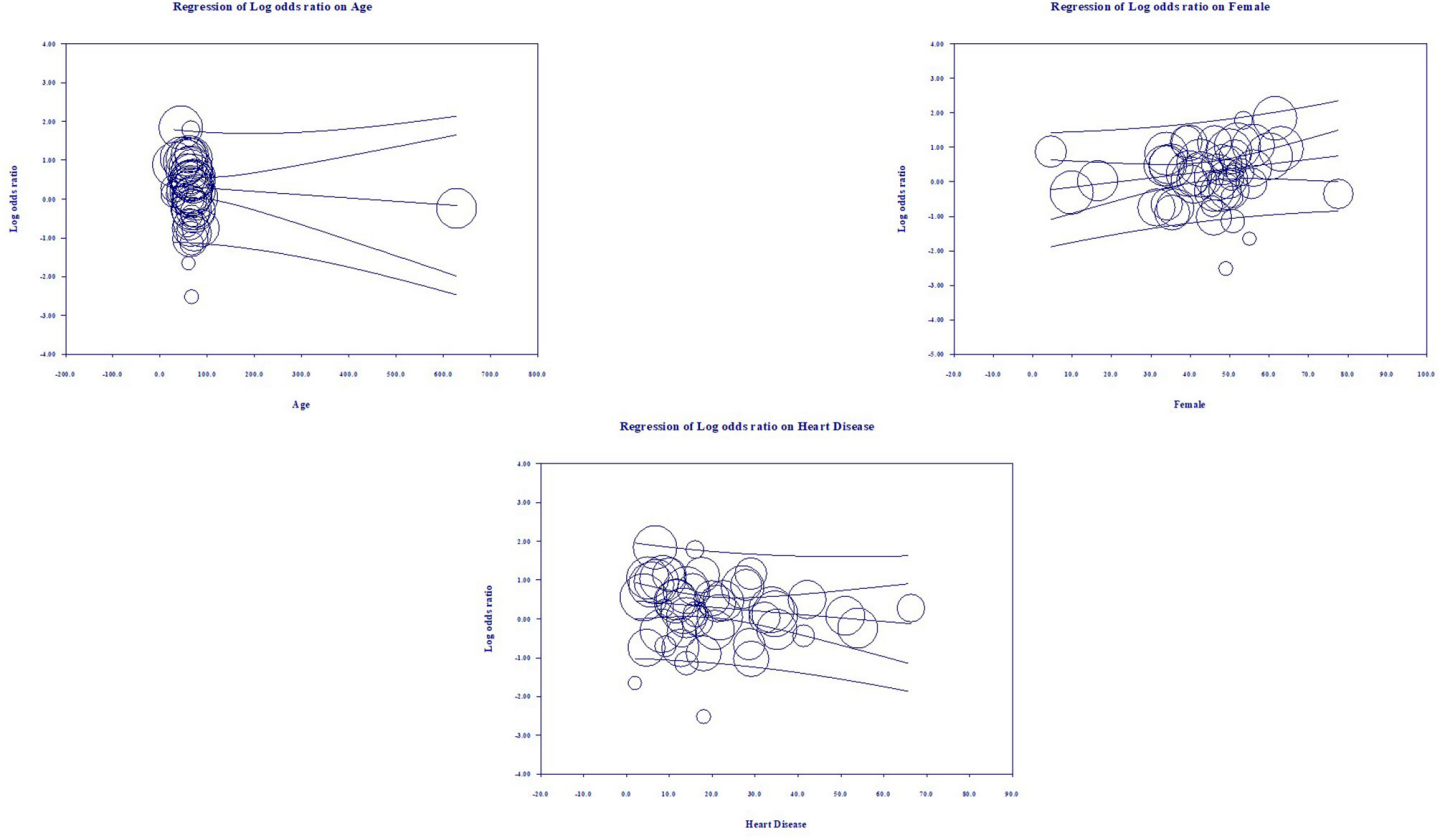
Meta-regression results for covariates significantly influencing mortality (*R*^2^ = 36).

Multivariate meta-regression performed to explain variations in association between mortality and being on ACEIs/ARBs revealed; age, female gender, proportion of heart diseases in included studies covariates to be significant together and explained *R*^2^ = 36% of the between study heterogeneity in mortality. Figure 4 shows the resulting equation and individual covariate effect graphs.

### Multivariate Meta-Regression Model for Severity Outcome

**Figure 5:**
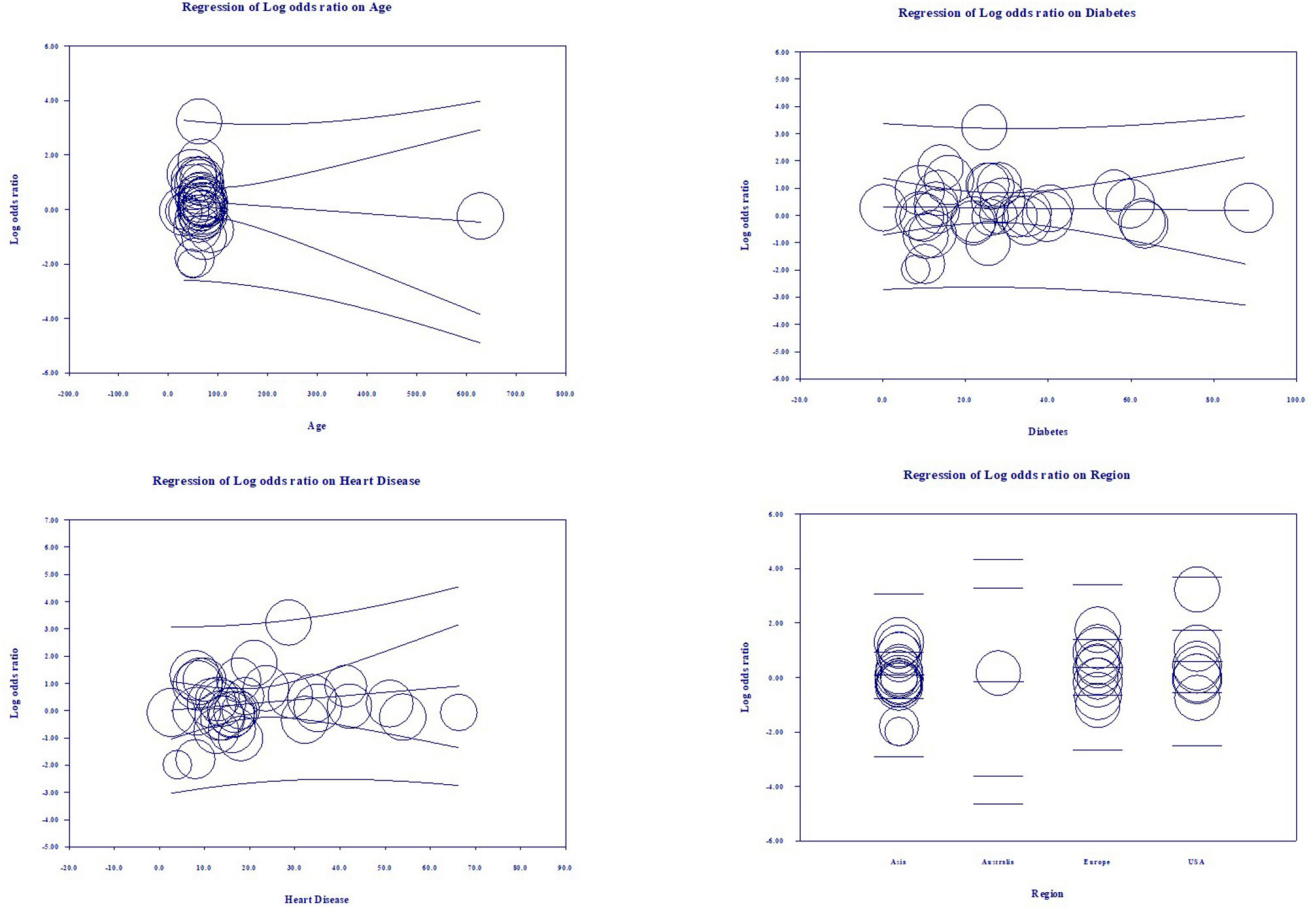
Meta-regression results for covariates significantly influencing severity (*R*^2^ = 8%).

Multivariate meta-regression performed to explain variations in association between severity and being on ACEIs/ARBs revealed age, proportion with diabetes, heart disease, and country of studies covariates to be significant together. These covariates together explained *R*^2^ = 8% of the study heterogeneity in severity. Figure 5 shows the resulting equation and individual covariate effect graphs.

### Publication Bias

**Figure 6:**
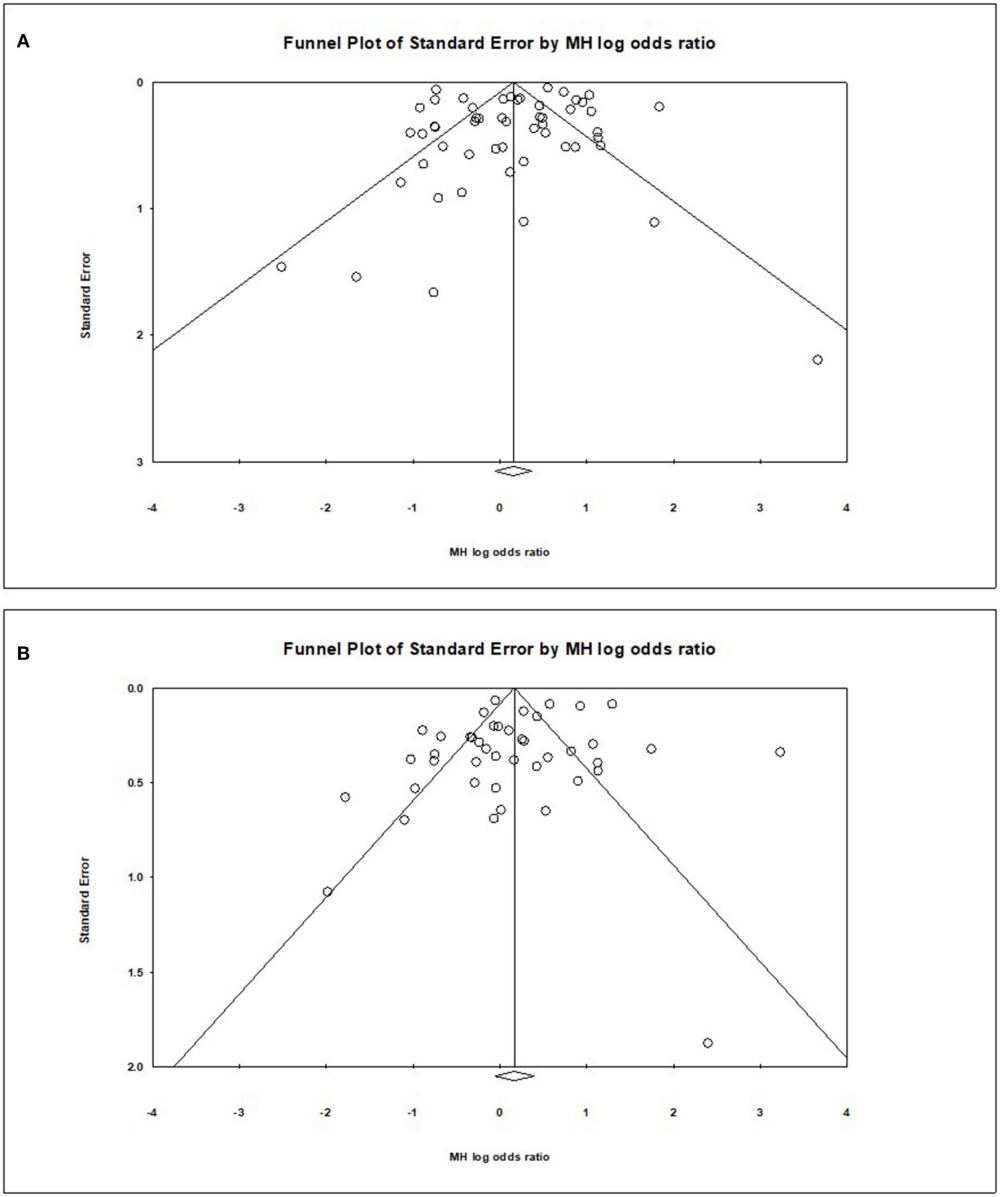
Funnel plots for publication bias of mortality **(A)** and severity **(B)** models.

Visual inspection of the standard error plots for the mortality analysis (Figure 6A) suggests symmetry without an underrepresentation of studies of any precision but indicated underrepresentation of studies with smaller effect sizes. Classic fail-safe N analysis computed taking alpha at 0.05 put the number of missing studies at 5. Corroborating inspection findings, in Egger's regression test the null hypothesis of no small study effects was not rejected at *P* < 0.05 (estimated bias coefficient = −0.28 ± 0.76 SE).

Similarly, visual inspection of the standard error plots for the severity analysis also (Figure 6B) suggest symmetry without an underrepresentation of studies of any precision but indicated underrepresentation of studies with smaller effect sizes. Classic fail-safe N analysis computed taking alpha at 0.05 put the number of missing studies at 8. However, in Egger's regression test the null hypothesis of no small study effects was rejected at *P* < 0.05 (estimated bias coefficient = −1.14 ± 0.81SE).

## Discussion

Based on our meta-analysis consisting of cross-sectional, case-control, and cohort studies, the use of ACEIs and ARBs was neither associated with increased all-cause mortality nor with increased severity of disease progression in COVID-19 patients. Multivariate meta-regression for the mortality model demonstrated that 36% of study variations could be explained by differences in age, female gender, proportion of heart diseases in the study samples. Multivariate meta-regression for the severity model demonstrated that 8% of study variations could be explained by differences in age, proportion of diabetes, heart diseases and country of studies in the study samples. This finding is valuable as association between ACEIs and ARBs use and outcome in COVID-19 patients has been inconclusive so far. To our knowledge, this is the first meta-regression, and the largest meta-analysis to evaluate the role of ACEIs/ARBs as an antihypertensive regimen in hospitalized patients with COVID-19.

The effect of ACEIs/ARBs use on COVID-19 patients has been a controversial topic since the beginning of this pandemic, and some studies even have interposed a risk of taking ACEIs/ARBs using data from previous coronavirus outbreaks and preclinical studies (99). Previously published systematic reviews suggested a lower mortality (25–43%) in patients with hypertension hospitalized for COVID19 (100–102). Furthermore, a large-scale retrospective study demonstrated that in-hospital use of ACEIs/ARBs was associated with a lower risk of 28-day death among hospitalized patients with COVID-19 and coexisting hypertension (adjusted HR 0.32, 95% CI 0.15–0.66) (80). These data suggested that patients with hypertension might obtain benefits from taking ACEIs/ARBs compared with the non-ACEIs/ARBs in the setting of COVID-19 and support the hypothesis that a drug that diminishes angiotensin-2 activity, such as ACEIs and/or ARBs, can reduce the deadliness of inflammation associated injury in COVID-19. Our result trends in contraindicating the above study results but did not reach statistical significance. Our meta-analysis suggests that the use of ACEIs/ARBs neither increase nor decrease mortality in COVID-19 patients (Figure 2). In addition to what is reported in published studies, our systematic review added the most recent studies, and had the largest sample size (53 studies, 112,468 patients).

The main strength of our analysis is the large sample size along with a robust and comprehensive search. The large sample size enables the precision and reliability of risk estimates. Additionally, further meta-regression was performed to adjust for confounding factors. However, despite all the strengths, there are still certain limitations. The major limitation of the meta-regression in the presence of unknown confounders. Multiple previous studies have reported that gender, age, smoking history, and presence of diabetes influence COVID-19 results. Even though these confounders are reported in most of the included studies, further studies focusing on the adjustment of confounders are necessary. We included studies from the medRxiv.org databases and other preprint databases which did not go through peer review at that time. We considered this as a limitation, as peer reviewers could catch more deficiencies in reporting methods and other details. However, it was anticipated that majority of these studies would be peer-reviewed. Third, the use of ACE/ARB has been via medical record review which could be less reliable. Fourth, there is a possibility of publication bias as the definition of COVID-19 severity and outcomes were not uniform among the included studies. Fifth, substantial clinical variability among COVID-19 patients throughout in included studies leads to a high degree of statistical heterogeneity in the analysis of COVID-19 mortality and severity. To overcome this, we did a meta-regression analysis to define heterogeneity in included studies. Sixth, we did not include racial or ethnicity variation as a covariant in meta-regression analysis. However, we included the country of study origin as a covariate in meta-regression to overcome this limitation. Lastly, since most of the patients in the study population were in a hospital, so the results may be subject to selection bias.

## Conclusion

Due to the lack of statistical significance in the meta-analysis and observed study variance in the meta-regression analysis, it is not reasonable to conclude that ACEIs and ARBs are either detrimental or beneficial for patients with COVID-19. Larger observational studies (3, 103–105) and clinical trials are warranted to confirm these findings. Providers should continue to manage patient hypertension as per current treatment guidelines (106, 107) and clinical judgement until more robust evidence can say otherwise.

## Data Availability Statement

The original contributions presented in the study are included in the article/supplementary material, further inquiries can be directed to the corresponding author/s.

## Author Contributions

RS and VB contributed equally in the defining the study outline and manuscript writing. Data review and collection done by AT, FA, HK, JM, KM, PG, RS, SA, and SR. Statistical analysis was done by AB, MS, and VB. Study design and critical review done by IM and RK. RS, VB, AB, and MS are the guarantor of the paper, taking responsibility for the integrity of the work as a whole, from inception to published article. All authors contributed to the article and approved the submitted version.

## Conflict of Interest

The authors declare that the research was conducted in the absence of any commercial or financial relationships that could be construed as a potential conflict of interest.

## Publisher's Note

All claims expressed in this article are solely those of the authors and do not necessarily represent those of their affiliated organizations, or those of the publisher, the editors and the reviewers. Any product that may be evaluated in this article, or claim that may be made by its manufacturer, is not guaranteed or endorsed by the publisher.
